# Supplementary material for: Artificial Intelligence-Based Opportunities in Liver Pathology—A Systematic Review
Source: Diagnostics (Basel). 2023 May 19;13(10):1799. doi: 10.3390/diagnostics13101799 (PMC10217251; doi:10.3390/diagnostics13101799)
Supplement: Supplementary file 1 [file diagnostics-13-01799-s001.zip › TABLE S2 models details.pdf]

Authors [reference]	DNN models tested (* marks the best model if multiple)
Feng Y. et al. [11]	*U-Net
	DeeplabV3
	GCN
	SegNet
	PSPNet
	DeconvNet
	Atten-Unet
	FCN
Cancian P. et al. [12]	U-Net
	SegNet
	*DeeplabV3
Roy M. et al. [13]	*HistoCAE (own)
	ResNet101
	Vgg19
	DenseNet
	InceptionV3
	U-Net
	SegNet
	DeeplabV3
	PSPNet
	RefineNet
	Mobile-UNet
Wang X. et al. [14]	*SEResNeXt-101
	SegNet
	DeeplabV3
	U-Net++
	Dupsample
	OCNet
Feng S. et al. [15]	ResNet18
Yang TL. et al. [16]	VGG16
	InceptionV4
	*FA-MSCN (own)
Li S. et al. [17]	MFC-CNN-softmax (own)
	*MFC-CNN-ELM (own)
Kiani A. et al. [18]	DenseNet

Schau GF. et al. [19]	own
Ercan C. et al. [20]	U-net with ResNet34
Diao S. et al. [21]	*MSAN-CNN (own)
Diao S. et al. [21]	U-Net++
Diao S. et al. [21]	DeeplabV3
Diao S. et al. [21]	Lite R-ASPP
Cheng N. et al. [22]	HnAIM (own)
Chen M. et al. [23]	InceptionV3
Liao H. et al. [24]	own
Saillard C. et al. [25]	*SCHMOWDER (own)
Saillard C. et al. [25]	CHOWDER (own)
Yamashita et al. [26]	HCC-SurvNet (own)
Saito A. et al. [27]	own
Xiao C. et al. [28]	ResNet-50
Chen Q. et al. [29]	MVI-DL (own)
Zeng Q. et al. [30]	patch-based model (own)
Zeng Q. et al. [30]	classic MIL model (own)
Zeng Q. et al. [30]	*CLAM model (own)
Qu WF. et al. [31]	InceptionV3
Xie J. et al. [32]	U-Net + AlexNet
Guo X. et al. [34]	Clump_seg
Guo X. et al. [34]	ResNet41
Guo X. et al. [34]	*ResNet50
Guo X. et al. [34]	ResNet65
Jirik M. et al. [35]	UNet-Mini (own)
Roy M. et al. [36]	Delineate/U-Net (own)
Yu H. et al. [37]	MUSA-Unet (own)
Vanderbeck S. et al. [38]	own
Vanderbeck S. et al. [39]	own
Wang TH et al. [40]	own
Munsterman I. et al. [41]	own
Klimov S. et al. [42]	NA
Puri M. [43]	AutoML Vision
Forlano R. et al. [44]	own
Teramoto T. et al. [45]	own
Salvi M. et al. [46]	HEPASS (own)

Gawrieh S. et al. [47]	own
Perez-Sans F. et al. [48]	K-Nearest Neighbors model
	*Naïve Baynes model
	Random Forest model
	Support Vector Machine model
	simple Neural Network model
	Keras model
Marti-Aguado D. et al. [49]	MATLAB (own)
Sjöblom N. et al. [50]	K7-AI
Ramkissoon R. et al. [51]	NA
Heinemann F. et al. [52]	InceptionV3
Constantinescu C. et al. [53]	own

NA Not Available
