# Supplementary material for: Artificial Intelligence-Based Opportunities in Liver Pathology—A Systematic Review
Source: Diagnostics (Basel). 2023 May 19;13(10):1799. doi: 10.3390/diagnostics13101799 (PMC10217251; doi:10.3390/diagnostics13101799)
Supplement: Supplementary file 1 [file diagnostics-13-01799-s001.zip › TABLE S4 QUADAS 2 detail.pdf]

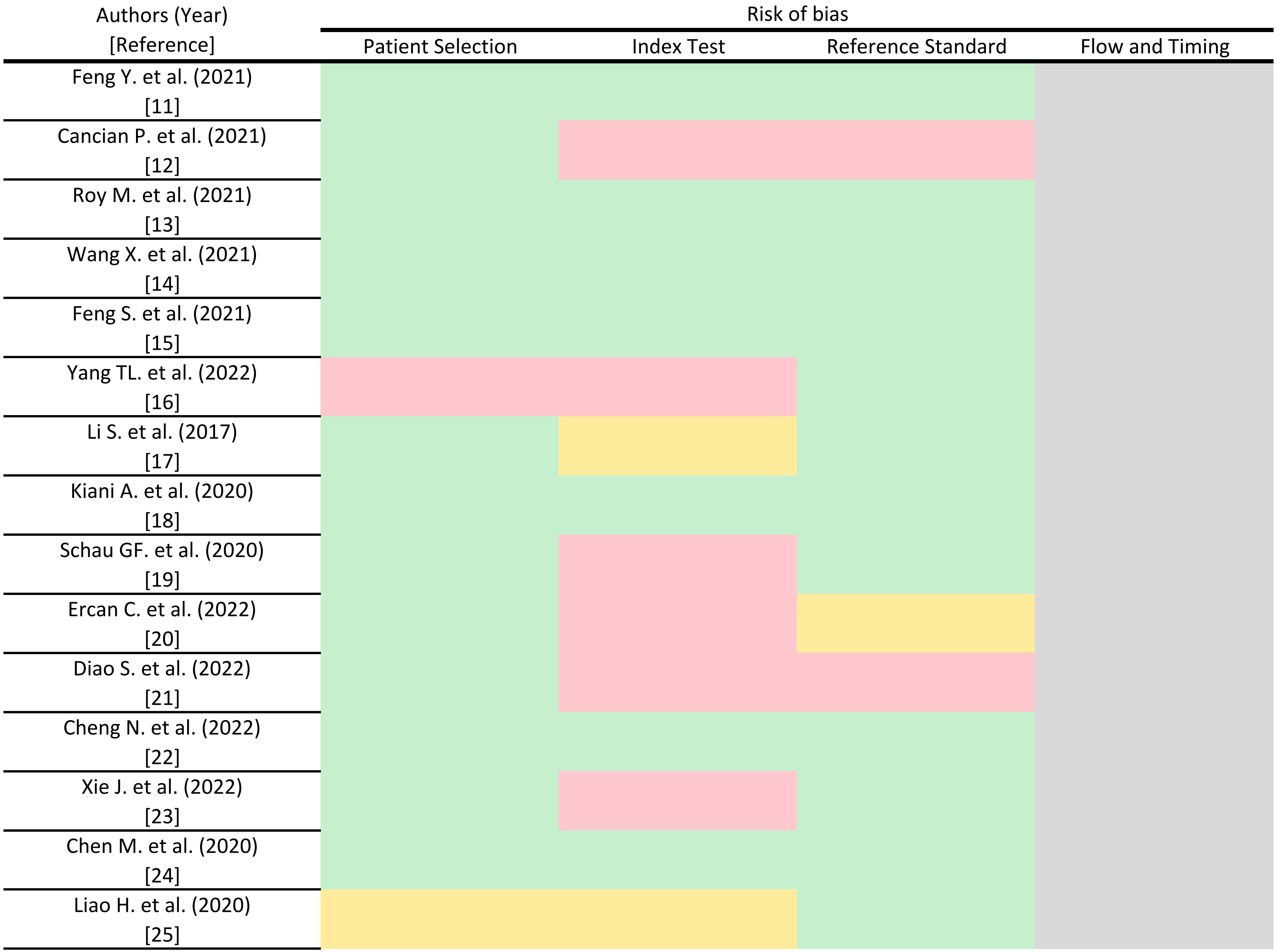
Authors (Year)
[Reference]
Risk of bias
Patient Selection
Index Test
Reference Standard
Flow and Timing
Feng Y. et al. (2021)
[11]
Cancian P. et al. (2021)
[12]
Roy M. et al. (2021)
[13]
Wang X. et al. (2021)
[14]
Feng S. et al. (2021)
[15]
Yang TL. et al. (2022)
[16]
Li S. et al. (2017)
[17]
Kiani A. et al. (2020)
[18]
Schau GF. et al. (2020)
[19]
Ercan C. et al. (2022)
[20]
Diao S. et al. (2022)
[21]
Cheng N. et al. (2022)
[22]
Xie J. et al. (2022)
[23]
Chen M. et al. (2020)
[24]
Liao H. et al. (2020)
[25]

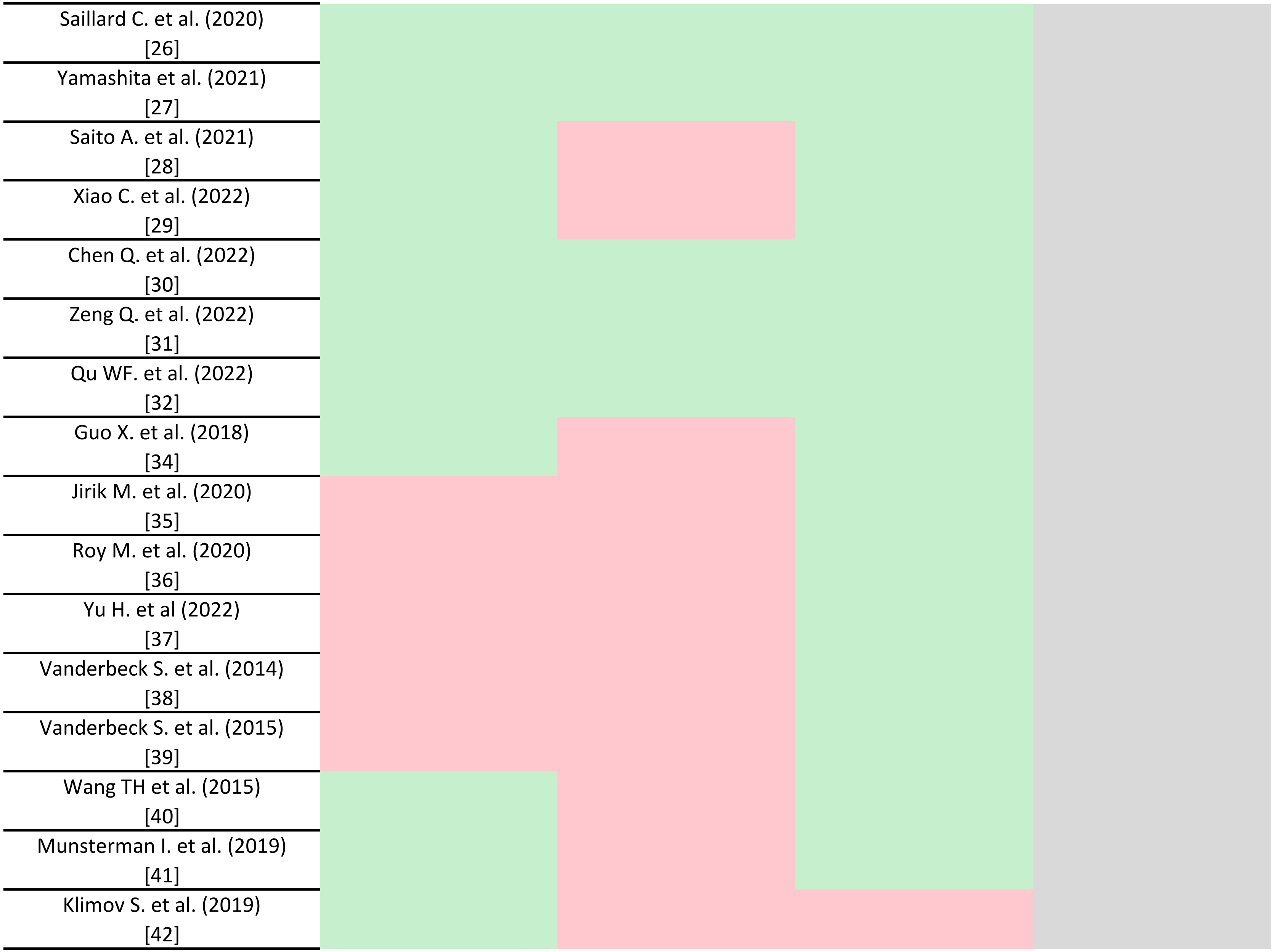
Saillard C. et al. (2020) [26]
Yamashita et al. (2021) [27]
Saito A. et al. (2021) [28]
Xiao C. et al. (2022) [29]
Chen Q. et al. (2022) [30]
Zeng Q. et al. (2022) [31]
Qu WF. et al. (2022) [32]
Guo X. et al. (2018) [34]
Jirik M. et al. (2020) [35]
Roy M. et al. (2020) [36]
Yu H. et al (2022) [37]
Vanderbeck S. et al. (2014) [38]
Vanderbeck S. et al. (2015) [39]
Wang TH et al. (2015) [40]
Munsterman I. et al. (2019) [41]
Klimov S. et al. (2019) [42]

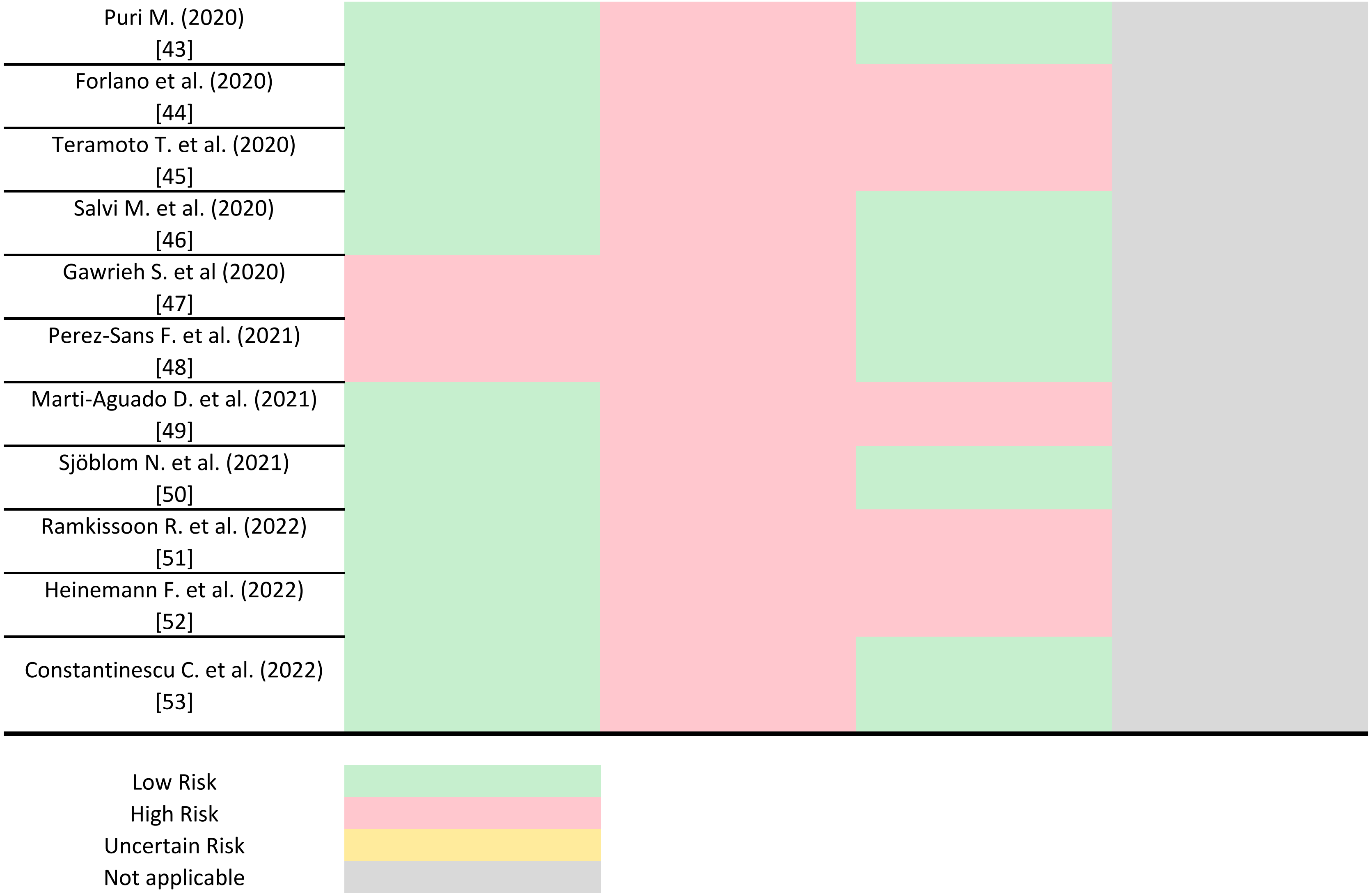
Puri M. (2020)
[43]
Forlano et al. (2020)
[44]
Teramoto T. et al. (2020)
[45]
Salvi M. et al. (2020)
[46]
Gawrieh S. et al (2020)
[47]
Perez-Sans F. et al. (2021)
[48]
Marti-Aguado D. et al. (2021)
[49]
Sjöblom N. et al. (2021)
[50]
Ramkissoon R. et al. (2022)
[51]
Heinemann F. et al. (2022)
[52]
Constantinescu C. et al. (2022)
[53]
Low Risk
High Risk
Uncertain Risk
Not applicable
